# Within-Person Dynamics of Job Boredom and Counterproductive Work Behavior: A Latent Change Score Modeling Approach

**DOI:** 10.1007/s42761-024-00256-y

**Published:** 2024-08-07

**Authors:** JeongJin Kim, Seth A. Kaplan, John A. Aitken, Lida P. Ponce

**Affiliations:** Department of Psychology, George Mason University, 4400 University Drive, Fairfax, VA 22030 USA

**Keywords:** Job boredom, Counterproductive work behavior, Within-person dynamics, Experience sampling method

## Abstract

Job boredom is one of the most common negative affective states experienced in the workplace, yet also among the least well-understood. One stream of research suggests that employees frequently react to job boredom by engaging in counterproductive work behaviors (CWB). However, recent studies show the converse—that engaging in CWB relates to job boredom. As studies on the job boredom-CWB relationship primarily have been cross-sectional and at the between-person level of analysis, the directionality between these constructs remains in question. Therefore, research examining the within-person dynamics of job boredom and CWB within a short timeframe is needed. In the current study, we explore whether job boredom influences subsequent changes in CWB and vice versa. We examined these relationships using latent change score (LCS) modeling with 10-day experience sampling data (*N* = 120 individuals providing 1,161 observations). Findings supported a reciprocal relationship. Employees’ level of job boredom on a given day was associated with a subsequent increase in CWB on the next day, and the level of CWB on a given day was associated with a subsequent increase in job boredom on the next day. We discuss the implications of our findings, study limitations, and future research directions.

Job boredom can be defined as an “unpleasant, transient affective state in which the individual feels a pervasive lack of interest in and difficulty concentrating on the current activity” (Fisher, 1993, p. 397). Job boredom is one of the most prevalent affective states in the workplace and relates to various harmful outcomes such as job dissatisfaction, turnover intentions, and mental health challenges (Fisher, 1993; Mael & Jex, 2015). Yet, job boredom also remains among the least understood forms of affect at work (Fisher, 1993; Loukidou et al., 2009).

In general, job boredom has been found to relate to increased counterproductive work behavior (CWB; e.g., Bruursema et al., 2011; Kim et al., 2021). CWB can be defined as voluntary behavior detrimental to the well-being of employees (e.g., gossiping, interpersonal conflict, aggression) and/or the organization (e.g., withdrawal, theft, cyberloafing) (Dalal et al., 2009). However, studies on job boredom and CWB primarily have been cross-sectional and at the between-person level (for an exception, see Spanouli et al., 2023), failing to yield insight into within-person dynamics of job boredom and CWB. Consequently, research is unclear about the directionality of the job boredom–CWB relationship at the within-person level of analysis. This lack of research is even more surprising given that CWB, along with job boredom, has been recognized as a transient and within-person phenomenon (McCormick et al., 2020; Podsakoff et al., 2019).

Therefore, we address this issue by exploring within-person reciprocal relationships between job boredom and CWB. Given that various (e.g., attentional/cognitive) theories make alternative directional predictions about the job boredom–CWB relationship, studying it in a within-person dynamic can speak to the correctness of these different theoretical ideas. Moreover, a small stream of research has begun examining the relationship of CWB with negative affect or other discrete negative emotions, and its directionality (e.g., Koopman et al., 2021). This study contributes to that stream of research by conducting a similar test on a negative discrete emotion that has been highlighted in the literature as a key predictor of CWB.

Research suggesting that job boredom predicts CWB mainly is based on Spector and Fox’s (2002) model of voluntary work behaviors. Consistent with this notion, studies demonstrate a positive link between job boredom and CWB, albeit at the between-person level (e.g., Bruursema et al., 2011; Kim et al., 2021; van Hooff & van Hooft, 2014). Research seems to support this relationship at the within-person level as well, as Spanouli et al. (2023) demonstrated a positive association between daily job boredom and CWB. As additional (though indirect) evidence, van Hooff and van Hooft (2017) showed that previous-day boredom related positively to current-day negative prework attitudes, which subsequently reduced intrinsic work motivation. Given the close within-person linkage between work attitudes/affect and CWB (Judge et al., 2006; Yang & Diefendorff, 2009), this mediation chain suggests that job boredom may lead to next-day increases in CWB. Thus, we expect current-day job boredom to relate positively to next-day increases in CWB (*Hypothesis 1*).

However, the reverse directionality may be plausible too: CWB may foster job boredom. This notion aligns with Tam et al.’s (2021) boredom feedback model, which posits that boredom emerges when there is inadequate attentional engagement, or a discrepancy between one’s desired and actual level of attentional engagement. CWB, as form of work disengagement, can foster boredom (which, in turn, can foster further CWB). Also, indirectly supporting the CWB to boredom notion, researchers have found that CWB tends to trigger other aversive states, including anger, frustration, guilt, and shame (e.g., Ilies et al., 2013; Spector & Fox, 2010).

This all said, boredom may not function analogously as those other emotions. Rather, it may be that CWB reduces (vs. enhances) job boredom. Functional theories of boredom posit that bored individuals attempt to regulate (or escape from) boredom by engaging in other tasks/activities that are more interesting or enjoyable than the current one (Bench & Lench, 2013, 2019; Elpidorou, 2018). Stated differently, engaging in CWB (e.g., cyberloafing; Pindek et al., 2018) could result in a lower level of subsequent job boredom. In sum, given the conflicting notions about the sign of this relationship, we do not offer a hypothesis, examining it in an exploratory manner.

## Method

### Participants and Procedure

Initially, we recruited 165 individuals who satisfied our eligibility criteria in the initial baseline survey. Of these, we excluded 12 participants who failed one or more of the three attention check items. We also omitted 33 participants who did not complete a minimum of two full days of daily surveys (i.e., four daily surveys). The final sample size was 120 individuals providing 1,161 observations (response rate = 92.49%). On average, participants were 32.94 years old (*SD* = 3.27) and worked an average of 36 hours per week (*SD* = 11.88). Most participants identified themselves as male (female = 34.17%), had held their jobs for 2–5 years (55.83%) or 5–9 years (41.67%), and had an occupation in business and finance (44.17%) or information and communication technology (22.50%). A majority of participants had obtained a bachelor’s degree (53.33%) and were either Black/African American (59.17%) or White (39.17%).

We recruited full-time employees by posting an advertisement on Craigslist, an online community website (see https://www.craigslist.org/about) in multiple large cities in the United States (see Judge et al., 2014; Vogel et al., 2020, for examples using Craigslist). The advertisement contained information about the study procedures, eligibility criteria, and compensation, and was posted for approximately two weeks in June 2022.

Interested participants were requested to email the researchers and were subsequently invited to complete an initial baseline survey, which included informed consent and measures of demographic variables. In this survey, participants were first screened based on three eligibility criteria: (a) age (i.e., 18 + years old), (b) full-time employment status (i.e., 30 + work hours per week), and (c) work schedules (i.e., whether they had a traditional work schedule from 9:00 AM to 5:00 PM), the last of which was employed to ease scheduling of daily surveys. At the end of the baseline survey, we employed a commitment device that asked participants to explicitly commit to completing a minimum of 75% of the daily surveys (Gabriel et al., 2019). If respondents failed to commit to completing 75% or more of daily surveys, we kindly asked them to consider increasing their commitment to maximize the utility of their effort in participating in the research study. As a result, all participants chose to commit 75% or more of the daily surveys.

For 10 consecutive workdays (i.e., two consecutive workweeks), participants received two brief surveys per day, the first survey at 10:00 a.m. (Time 1 or T1) and the second survey at 1:00 p.m. (Time 2 or T2). In both surveys, we assessed momentary measures of job boredom and CWB. In exchange for participation, we provided a maximum possible amount of 55.00 U.S. dollars in the form of an Amazon gift card.

To ensure data quality, we followed best practice recommendations for convenience sampling. Specifically, we implemented such strategies as posting the advertisement in major metropolitan areas (Antoun et al., 2016), including attention check items in the initial survey (e.g., “Please select *Never* for this item.”; Meade & Craig, 2012), and tracking unique identifiers to assure only one survey was completed per participant (Mason & Suri, 2012). Additionally, to ensure that our study had sufficient statistical power, we followed Gabriel et al.’s (2019) sample size recommendations, which were based on their calculation of the mean sample size of the past experience sampling studies. Accordingly, we aimed to obtain a minimum Level 2 sample size of 83 and a minimum Level 1 sample size of 835 by administering three daily surveys for 10 consecutive workdays. This strategy of sampling for two weeks allowed us to capture a generally representative sample of a person’s daily experiences (Wheeler & Reis, 1991).

### Measures

#### Daily Job Boredom

Momentary job boredom was assessed using a 4-item measure (Park et al., 2019). On a 5-point Likert scale (1 = *not at all* to 5 = *extremely*), participants were asked to rate the extent to which they were feeling each of the following four emotion adjectives at the current moment: “bored,” “sluggish,” “dull,” and “lethargic.” Given our interest in studying the job boredom–CWB relationship at the daily level of analysis, we created a daily measure of job boredom by aggregating job boredom assessed in the morning (T1) and in the afternoon (T2). Across the measurements, the average Cronbach’s alpha coefficient was .99. We chose to focus on the day level to capture the series of affective and performance episodes that transpire during a workday (see Weiss & Merlo, 2020). Aggregating lower-level ratings to a higher level (here, the day level) is a common practice in multilevel research, as doing so can help minimize recollection errors while also increasing reliability (Cortina et al., 2020; Gabriel et al., 2019).

#### Daily Counterproductive Work Behavior

CWB was measured with 2 items from Dalal et al., (2009). Although the full scale originally contained six items, we only included two of them that may be likely to have greater within-person variability during one’s typical workday (Koopman et al., 2021). On a dichotomous scale (1 = *yes*, 0 = *no*), we asked participants to rate each item while thinking about the last two hours at work. Example items include “Did not work to the best of my ability” and “Spent time on tasks unrelated to work.” As done with the measure of job boredom (based on the same justifications), we created a daily measure of CWB by aggregating CWB assessed in T1 and T2. Across the days of data collection, the average Cronbach’s alpha coefficient was .91.

### Data Analysis

**Fig. 1:**
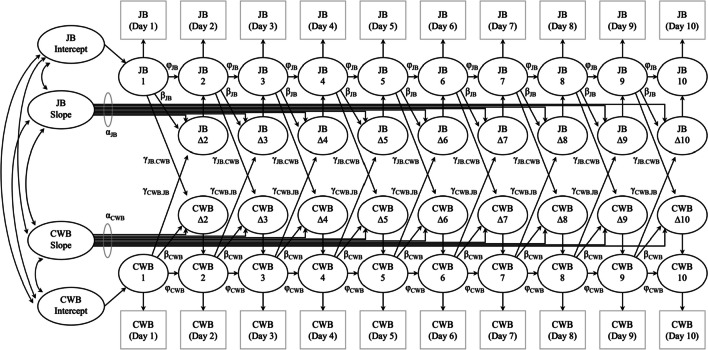
A proposed latent change score model for job boredom and counterproductive work behavior. *Note*. Adapted from Matusik et al. (2021). *JB* = job boredom; *CWB* = counterproductive work behavior; *α*_*x*_ represents basis coefficients (traditionally fixed to 1.0) for variable *x*; *φ*_*x*_ represents autoregression of change scores for variable *x*; *β*_*x*_ represents proportional change effect for variable *x*; *γ*_*JB.CWB*_ represents lagged coupling effect of job boredom on change in CWB; *γ*_*CWB.JB*_ represents lagged coupling effect of CWB on change in job boredom

To study within-person time-sequential relationships (i.e., dynamic “couplings,” or cross-lagged relationships) between job boredom and CWB, we used latent change score (LCS) modeling (see Matusik et al., 2021, for a review). As an unconditional/null model (or the “no coupling” model), we used 10 daily measurements of both job boredom and CWB and specified constant change effect (i.e., whether and to what extent there is an overall increasing or decreasing trend for a given variable), proportional change effect (i.e., whether and to what extent a level of the variable influences subsequent change in that same variable), and autoregression of change scores for both job boredom and CWB. In our final model (or the “full coupling” model), we additionally specified a lagged coupling effect of job boredom on Day *i* – 1 on change that occurs in CWB between Day *i* – 1 and Day *i*, as well as a lagged coupling effect of CWB on Day *i* – 1 on change that occurs in job boredom between Day *i* – 1 and Day *i*. The lagged coupling effects were the parameters of primary interest in this study. For instance, the lagged coupling effect of job boredom on change in CWB indicates whether and to what extent the latent true score of job boredom at Day *i* – 1 relates to increases (i.e., indicated by a significant, positive effect) or decreases (i.e., indicated by a significant, negative effect) in the latent change in CWB between Day *i* – 1 and Day *i*. As is common when using LCS models, all estimates were assumed to be equal across time points (Matusik et al., 2021). Figure 1 depicts our final bivariate LCS model.

## Results

Our final bivariate LCS model provided an acceptable fit (χ^2^(207) = 449.35, *p* < .001, CFI = .90, TLI = .91, RMSEA = .09, SRMR = .10). We first examined whether the final model fits the data significantly better than three alternative (nested) models, including the no coupling model and two unidirectional coupling models. In each of those two unidirectional coupling models, the lagged coupling parameter of one construct (e.g., job boredom or CWB) on change in the other construct (e.g., CWB or job boredom) was added to the no coupling model. The no coupling model provided a moderate fit (χ^2^(209) = 486.75, *p* < .001, CFI = .87, TLI = .89, RMSEA = .10, SRMR = .11) and provided a worse fit than the final model (Δχ^2^(2) = 11.46, *p* = .003). The first univariate coupling model, in which the lagged effects of job boredom on changes in CWB were freely estimated, also showed a moderate fit (χ^2^(208) = 485.12, *p* < .001, CFI = .88, TLI = .89, RMSEA = .10, SRMR = .10) and provided a worse fit than the final model (Δχ^2^(1) = 28.16, *p* < .001). Finally, the second univariate, in which the lagged effects of CWB on changes in job boredom were freely estimated, showed a moderate fit (χ^2^(208) = 474.25, *p* < .001, CFI = .88, TLI = .89, RMSEA = .10, SRMR = .11) and provided a worse fit than the final model (Δχ^2^(1) = 6.44, *p* = .011). In sum, the final, bivariate full coupling model showed significant improvements in fit over all three alternative models and therefore was retained as the final model.

Turning to the primary study questions, we first report results regarding constant change (i.e., µ) to examine whether there was a significant overall trend, and then report findings regarding lagged coupling effects (i.e., γ). As reported in Table 1, results showed that, regarding job boredom, the constant change estimate was not statistically significant (µ = –1.36, *p* = .289). The nonsignificant constant change estimate indicates that there was no general increasing or decreasing trend in job boredom over the course of 10 workdays. Regarding CWB, findings showed that the constant change effect was significant and positive (µ = .78, *p* = .030), indicating that there was a general increasing trend in CWB engagement over the course of 10 workdays.

**Table 1:** Parameter estimates for the proposed latent change score model examining the within-person dynamics of job boredom and counterproductive work behavior

Parameter		Estimate (*SE*)	*p*
Latent true score mean			
	µ_JB0_	1.71 (.07)	< .001
	µ_CWB0_	1.17 (.02)	< .001
Constant change effect			
	µ_JB1_	–1.36 (1.28)	.289
	µ_CWB1_	.78 (.36)	.030
Proportional change effect			
	β_JB_	–1.29 (.32)	< .001
	β_CWB_	–1.11 (.43)	.010
Autoregression of change scores			
	φ_JB_	.42 (.13)	.001
	φ_CWB_	1.27 (.21)	< .001
Lagged coupling effect			
	γ_JB.CWB_	.32 (.15)	.039
	γ_CWB.JB_	2.95 (1.26)	.020

*JB* job boredom, *CWB* counterproductive work behavior, *γ*_*JB.CWB*_ latent change in JB between Day *i* – 1 and Day *i* determined by latent true score of CWB at Day *i* – 1, *γ*_*CWB.JB*_ latent change in CWB between Day *i* – 1 and Day *i* determined by latent true score of JB at Day *i* – 1

Next, we report findings regarding lagged coupling effects. Results showed the lagged coupling effects of job boredom on subsequent changes in CWB was significant and positive (γ = .32, *p* = .039). That is, true score values for job boredom at Day *i* – 1 were positively associated with change score values for CWB at Day *i*. Consistent with our prediction, this suggests that employees who experience greater job boredom on a given day are likely to engage in more CWB on the next day, compared to those who are less bored. Therefore, findings provide support for Hypothesis 1.

Regarding the reverse-direction relationship, findings also demonstrated that the lagged coupling effect of CWB on subsequent changes in job boredom was significant and positive (γ = 2.95, *p* = .020). That is, true score values for CWB at Day *i* – 1 were positively associated with change score values for job boredom at Day *i*. This suggests that employees who engage in more CWB on a given day are likely to experience greater job boredom on the next day, compared to those who engage in less CWB.

## Discussion

In this study, we examined within-person changes in job boredom and CWB over a short timeframe. Using LCS modeling, we found that reciprocal within-person relationships exist between job boredom and CWB. Consistent with our prediction, we found that job boredom related positively to next-day CWB change. We also found a positive relationship between CWB and next-day boredom change, suggesting that CWB enactment may increase boredom rather than reducing it. Although this latter finding is inconsistent with the theorized regulatory function of boredom, it is consistent with Tam et al.’s (2021) boredom feedback model suggesting that attentional disengagement leads to boredom, and also consistent with results showing that CWB engagement leads to other negative affective states (e.g., shame, guilt, frustration, anger; Ilies et al., 2013; Spector & Fox, 2010).

Our results extend the literature on job boredom and CWB by providing empirical evidence for within-person change in job boredom and CWB over a short timeframe. Most studies on job boredom have used cross-sectional designs and are at the between-person level of analysis. Using cross-lagged data in the experience sampling framework, the current study provides evidence clarifying the directionality of the within-person relationship between job boredom and CWB. In addition, our findings contribute to the stream of research testing the directionality of relationships between negative affect/discrete emotions and CWB (e.g., Koopman et al., 2021).

Regarding practical implications, our findings suggest that HR practitioners should be cognizant that CWB enactment may be generative, not reparative, of job boredom. As recent studies show beneficial effects of some CWB forms on employee well-being, such as cyberloafing (e.g., Lim & Chen, 2012), a reasonable inference is that such activities may mitigate boredom. However, the current results suggest that the opportunity to engage in such activities could instead foster (more) boredom. In this sense, those activities may trigger the outcomes they partly are meant to avoid. In general, more research is needed on organizational policies and interventions meant to mitigate job boredom.

Moreover, our findings indirectly suggest a potential cycle, in that job boredom on a given day increases CWB on the next day, which increases job boredom on the following day, and so forth. This is consistent with cognitive/attentional models of boredom. Attentional models suggest that, when either the intention to attend or cognitive resources are absent, people experience feelings of boredom and subsequently lose engagement in tasks (Eastwood et al., 2012; Tam et al., 2021). The current study was unable to test this possibility directly, though, and so future research studying this idea would be useful.

As with other studies, the current study has limitations. The first limitation pertains to our sample. Because we sampled participants from an online community website (Craigslist), we carefully recruited participants based on multiple eligibility criteria and excluded some participants from data analysis based on a priori inclusion criteria. Despite the supportive views in applied psychology toward convenience sampling and online panel data (Landers & Behrend, 2015; Walter et al., 2019), one may still question the external validity of findings given this sampling strategy and resultant data. Therefore, replicating and extending our findings using more conventionally sourced data (e.g., organizational data) would be helpful.

Another limitation involves data aggregation regarding the measures of job boredom and CWB. Our data aggregation was based on theoretical views of episodic affect and performance (Weiss & Merlo, 2020). Also, from a measurement perspective, aggregating momentary measures to a daily measure is superior to simply asking participants to recall their levels of boredom and CWB over the whole day (Beal, 2015). However, one limitation is that the daily sample consisting of only two momentary measures (one in the morning and the other in the afternoon) may not be a complete aggregate (e.g., Sliwinski, 2008). Therefore, capturing more momentary measures of boredom or CWB would be helpful.

Lastly, given the wording of the two items used to assess CWB in this study (viz., “Did not work to the best of my ability” and “Spent time on tasks unrelated to work”), the measurement of CWB in this study could be seen as a more attentional or disengagement measure (i.e., whether people are focusing on their work) versus a behavioral measure. As such, replicating these findings with other CWBs (measures) would seem useful.

## Additional Information

### Data Availability

The dataset used in the current study is available in the Open Science Framework repository, https://osf.io/439nc/?view_only=1ffbb172575049939e1d3b1f3e134836.
